# Prognostic and clinicopathological value of systemic immune-inflammation index in patients with osteosarcoma: a meta-analysis

**DOI:** 10.3389/fimmu.2024.1416068

**Published:** 2024-08-15

**Authors:** Xiaoyan Wang, Zhong Wu, Zongxin Zhang, Ziwei Jiang

**Affiliations:** ^1^ Clinical Laboratory, Huzhou Central Hospital, Affiliated Central Hospital of Huzhou University, Huzhou, Zhejiang, China; ^2^ Clinical Laboratory, Nanxun District Hospital of Traditional Chinese Medicine, Huzhou, Zhejiang, China; ^3^ Clinical Laboratory, People’s Hospital of Anji, Huzhou, Zhejiang, China

**Keywords:** systemic immune-inflammation index, meta-analysis, osteosarcoma, evidence-based medicine, prognosis

## Abstract

**Background:**

The efficiency of systemic immune-inflammation index (SII) in predicting prognosis of osteosarcoma (OSA) patients has been extensively analyzed, but no consistent findings are obtained. Therefore, this meta-analysis focused on identifying the precise prognostic value of SII for OSA.

**Methods:**

We comprehensively searched electronic databases of PubMed, Embase, Web of Science, Cochrane Library, and China National Knowledge Infrastructure (CNKI) from inception to 24 February, 2024. Meanwhile, the efficiency of SII in predicting prognosis of OSA was evaluated by calculating pooled hazard ratios (HRs) as well as 95% confidence intervals (CIs). Additionally, the correlation of SII with the OSA clinicopathological characteristics was analyzed based on pooled odds ratios (ORs) and 95%CIs.

**Results:**

Six studies with 1015 cases were enrolled into this work. According to the combined data, the higher SII was markedly related to poor overall survival (OS) (HR=2.01, 95%CI=1.30-3.09, p=0.002) and Enneking stage III (OR=2.21, 95%CI=1.11-4.39, p=0.024) of patients with OSA. Nonetheless, SII was not significantly related to gender, age, pathological fracture, tumor size, tumor location, tumor differentiation, and metastasis in patients with OSA.

**Conclusions:**

In summary, the higher SII is markedly related to poor OS and advanced Enneking stage in OSA patients.

**Systematic review registration:**

https://inplasy.com/inplasy-2024-7-0107/,

identifier INPLASY202470107.

## Introduction

Osteosarcoma (OSA) shows the highest morbidity among primary malignant bone tumor among children and young adults (1). It exhibits the typical feature of formation of immature osteoid by tumor cells (2). All OSA patients have a median age of 20 years. The incidence of OSA in children and young adults worldwide varies between 2-3 per million, accounting for about 20%-30% of all primary bone tumors (3). In the past several decades, OSA patients have been treated by multiple strategies like surgery, radiotherapy, chemotherapy, gene therapy and immunotherapy (4). In spite of this, the prognosis of OSA remains poor. The 5-year survival rate in patients with localized OSA is about 60%, but that is only 20% in those developing metastases or recurrent disease (5). Around 10-15% of newly diagnosed OSA cases develop metastases, usually located in the lung (6). Therefore, it is of urgent necessary to develop novel and creditable markers for predicting prognosis of patients with OSA.

It has been widely reported that cancer-related inflammation and immune system have a critical effect on carcinogenesis, tumor growth, progression and metastasis (7, 8). Many hematological parameters including platelet-to-lymphocyte ratio (9), lymphocyte-to-monocyte ratio (10), prognostic nutritional index (PNI) (11), neutrophil-to-lymphocyte ratio (12), and systemic immune-inflammation index (SII) (13), are identified as efficient markers for predicting prognosis of various cancers. SII was developed in 2014 and reflects the general immune status of the body as an easy-to-access inflammatory parameter (14). SII is calculated as follows: SII= platelet number* neutrophil number/lymphocyte number (14). Notably, SII is previously reported to be significant for predicting prognosis of diverse solid tumors including rectal cancer (15), non-small cell lung cancer (16), breast cancer (17), renal cell carcinoma (18), and pancreatic neuroendocrine tumors (19). Meanwhile, the efficiency of SII in predicting prognosis of OSA patients is widely analyzed in numerous studies, but no consistent findings are obtained (20–25). For example, in some studies, the higher SII significantly predicted the prognosis of OSA patients (21, 22, 24, 25). Whereas in others, SII is not markedly related to the prognosis of OSA patients (23). Consequently, the present work was performed for identifying the accurate role of SII in predicting prognosis of OSA patients. Moreover, we investigated the correlation of SII with the OSA clinicopathological factors.

## Materials and methods

### Study guideline

The present work was conducted following the Preferred Reporting Items for Systematic Reviews and Meta-Analyses (PRISMA) guideline (26). This meta-analysis was registered in INPLASY under the registration number INPLASY202470107. This protocol can be available at https://inplasy.com/inplasy-2024-7-0107/.

### Ethics statement

Ethical approval and informed consent were waived since the present work was conducted using previously published data.

### Literature search

PubMed, Embase, Web of Science, Cochrane Library, and China National Knowledge Infrastructure (CNKI) databases were thoroughly retrieved between inception and 24 February, 2024 using the following search strategies (systemic immune-inflammatory index OR systemic-immune-inflammation index OR systemic immune-inflammation index) AND (osteosarcoma OR osteogenic sarcoma OR bone sarcoma). There was no limitation to publication language. The detailed search strategies for each database were shown in **Supplementary File 1**. Besides, we manually screened references in enrolled articles to identify more eligible studies.

### Eligibility criteria

Studies conforming to criteria below were included: (1) the diagnosis of OSA was made pathologically; (2) the relation of SII with prognosis of OSA was reported; (3) hazard ratios (HRs) and 95% confidence intervals (CIs) were available or calculable using Tierney’s method (27); (4) the SII threshold was provided; and (5) there was no restriction of publication language. Studies below were excluded: (1) meeting abstracts, reviews, case reports, comments, and letters; (2) those did not provide survival data; and (3) animal studies.

### Data collection and quality evaluation

Data were collected from qualified articles by two researchers (ZW and ZZ). Any disagreement between them was settled through discussion with a third researcher (ZJ) to reach a consensus. Data below were collected: first author, year, country, sample size, gender, age, study duration, study design, study center, Enneking stage, treatment, SII threshold, survival endpoints, survival analysis types, follow-up, and HRs with 95%CIs. Two independent reviewers (XW and ZZ) used Newcastle–Ottawa scale (NOS) to evaluate study quality (28). Notably, NOS evaluates study quality from 3 domains, selection, comparability, and outcome ascertainment. The total NOS score is 0-9 points, with ≥6 points indicating high-quality studies.

### Statistical analysis

The value of SII in predicting prognosis of OSA was analyzed by calculating combined HRs and 95%CIs. Moreover, the heterogeneity degree among enrolled studies was quantified by Cochran’s Q-test and Higgins I^2^ statistic. In the presence of obvious heterogeneity (I^2^>50% or p<0.010), we utilized a random-effects model; or else, we adopted a fixed-effects model. Additionally, the significance of SII for predicting prognosis of different subgroup OSA populations was explored by subgroup analysis. The correlation between SII and the OSA clinicopathological factors was investigated through pooling odds ratios (ORs) and 95%CIs. In the meantime, sensitivity analysis was also conducted for comparing the pooled effect when each study was excluded individually to determine whether a particular study is responsible for heterogeneity and to ensure results are stable. Publication bias was evaluated by using Begg’s funnel plot and Egger’s test. Stata version 12.0 (Statacorp, College Station, TX, USA) was employed for statistical analysis. p<0.05 stood for statistical significance.

## Results

### Process of literature search

**Figure 1:**
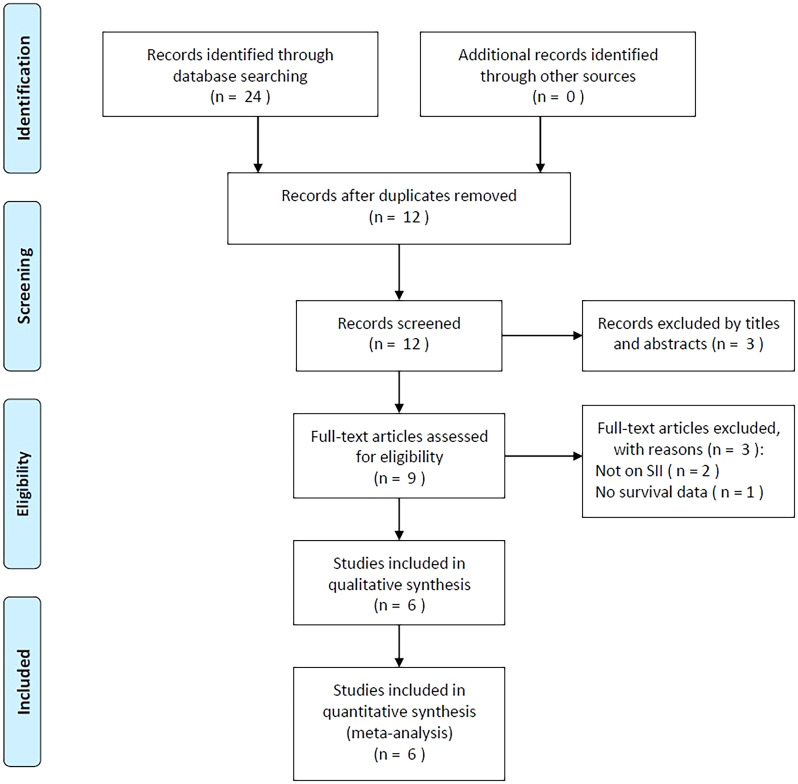
Flow diagram of study selection.

Initially, 24 studies were obtained through primary retrieval, among which, 12 were maintained when duplicates were eliminated (**Figure 1**). By title and abstract screening, we discarded three articles due to irrelevance. Full-texts of the rest 9 studies were examined, among which, three were eliminated due to irrelevance of SII (n=2) and unavailable survival data (n=1). Finally, six studies with 1015 patients (20–25) were enrolled into this work (**Figure 1**; **Table 1**).

**Table 1:** Basic characteristics of included studies in this meta-analysis.

Study	Year	Country	Sample size	Gender (M/F)	Age (years) Median(range)	Study period	Study center	Enneking stage	Treatment	Survival outcomes	Survival analysis	Follow-up (months) Median(range)	NOS
Huang, X.	2019	China	126	78/48	30(6-72)	2012-2018	Single center	I-III	Mixed	OS	Multivariate	44(7-81)	7
Yang, S.	2020	China	77	43/34	19(7-66)	2010-2013	Single center	I-III	Mixed	OS	Univariate	1-100	7
Ma, C.	2022	China	125	84/41	31(6-80)	2012-2019	Multicenter	I-III	Mixed	OS	Multivariate	1-120	9
Yang, Y.	2022	China	487	283/204	20	2008-2018	Single center	II-III	Mixed	OS	Multivariate	To Dec 2020	7
He, H.	2023	China	92	51/41	28(11-66)	2000-2018	Multicenter	I-III	Mixed	OS	Univariate	58(37-66)	8
Wu, Y.	2023	China	108	61/47	34(9-67)	2012-2017	Single center	I-III	Mixed	OS	Multivariate	62(4-132)	8

M, male; F, female; OS, overall survival; NOS, Newcastle-Ottawa Scale.

### Enrolled study features

**Table 1** displays basic study features (20–25). These studies were published during 2019-2023 and were all conducted in China. There were four (20–23) and two (24, 25) studies published in English and Chinese separately. All included studies were of retrospective design (20–25). The sample size was 77-487 (median, 116.5). There were four single center studies (20, 21, 23, 25) and two multicenter studies (22, 24). Five articles enrolled OSA patients of Enneking stage I-III (20–22, 24, 25), while one included those of Enneking stage II-III (23). All studies treated patients with mixed treatments including surgery, chemotherapy, and radiotherapy (20–25). The median SII threshold was 639.48 (range, 384.9-869.04). Each of our enrolled articles mentioned the relation of SII with overall survival (OS) in patients with OSA. In four articles, the HRs and 95%CIs were obtained through multivariate analysis (20, 22, 23, 25), while in another two articles, they were acquired through univariate analysis (21, 24). NOS scores ranged from 7 to 9, suggesting high-quality studies (**Table 1**).

### SII and OS

**Figure 2:**
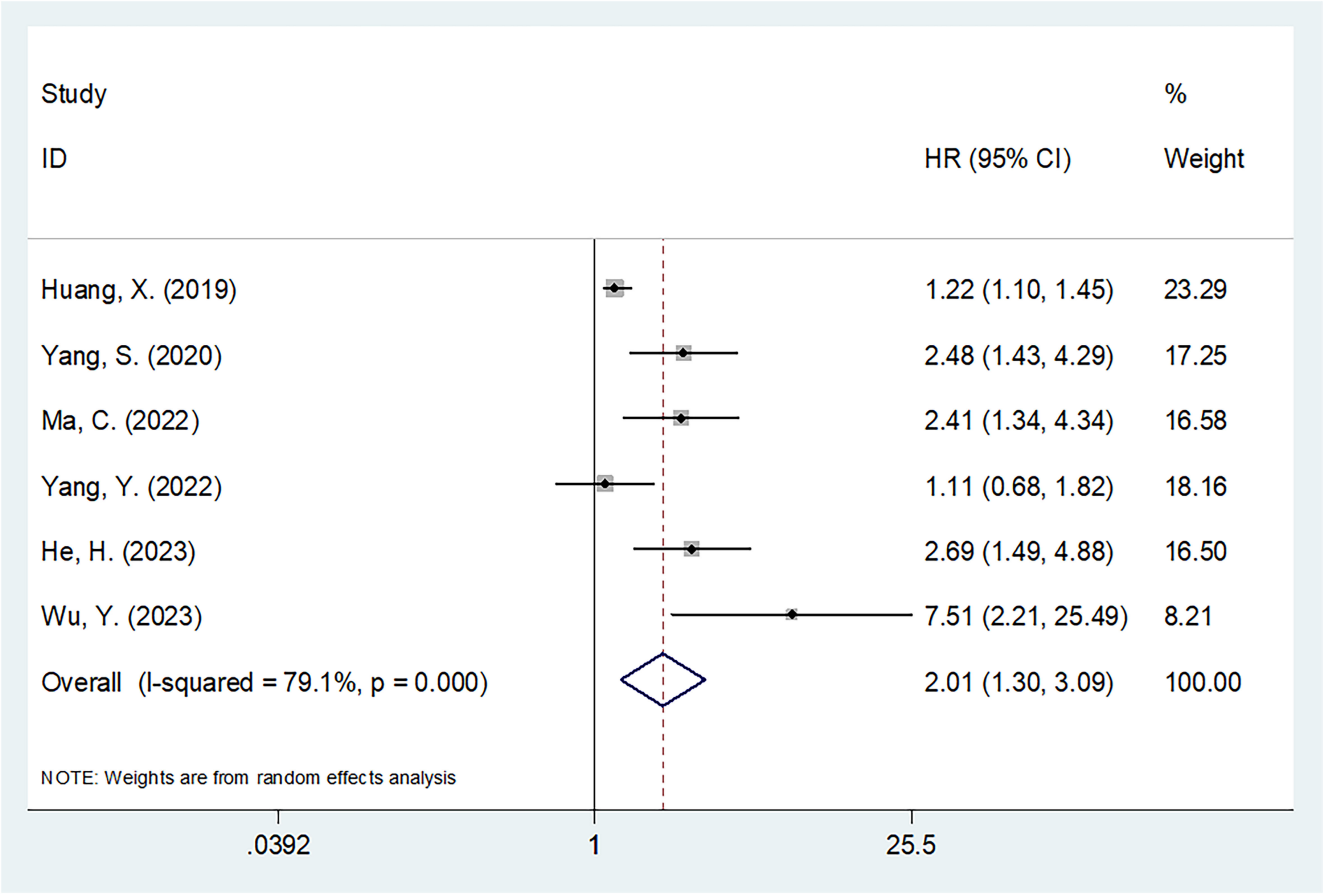
Forest plot for the prognostic value of SII for overall survival in patients with OSA.

The six articles involving 1015 patients (20–25) reported the value of SII in predicting OS of OSA patients. Due to obvious heterogeneity (I^2 =^ 79.1%, p<0.001), we used the random-effects model. HR=2.01, 95%CI=1.30-3.09, and p=0.002 were obtained from pooled results, suggesting that the higher SII was markedly related to dismal OS of OSA patients (**Table 2**; **Figure 2**). Upon subgroup analysis, SII still significantly predicted OS of OSA patients, irrespective of study center, threshold, and survival analysis types (p<0.02; **Table 2**). Additionally, SII was significantly related to dismal OS of OSA in subgroups below: sample size <120 (p<0.001) and patients with Enneking stage I-III (p=0.002) (**Table 2**).

**Table 2:** Subgroup analysis of the prognostic value of SII for OS in patients with osteosarcoma.

Subgroups	No. of studies	No. of patients	Effects model	HR (95%CI)	p	Heterogeneity	
						I2(%)	Ph
Total	6	1015	Random	2.01(1.30-3.09)	0.002	79.1	<0.001
Sample size							
<120	3	277	Fixed	2.86(1.95-4.19)	<0.001	25.7	0.260
≥120	3	738	Random	1.39(0.96-2.00)	0.080	61.3	0.075
Study center							
Single center	4	798	Random	1.78(1.05-3.02)	0.032	79.1	0.002
Multicenter	2	217	Fixed	2.55(1.68-3.87)	<0.001	0	0.796
Enneking stage							
I-III	5	528	Random	2.36(1.37-4.06)	0.002	82.7	<0.001
II-III	1	487	–	1.11(0.67-1.82)	0.685	–	–
Cut-off value							
<650	3	359	Random	2.33(1.01-5.36)	0.047	84.6	0.002
≥650	3	656	Random	1.92(1.08-3.42)	0.027	70.4	0.034
Survival analysis							
Univariate	2	169	Fixed	2.58(1.72-3.85)	<0.001	0	0.839
Multivariate	4	846	Random	1.76(1.04-2.95)	0.034	77.4	0.004

### The association of SII with the OSA clinicopathological features

**Figure 3:**
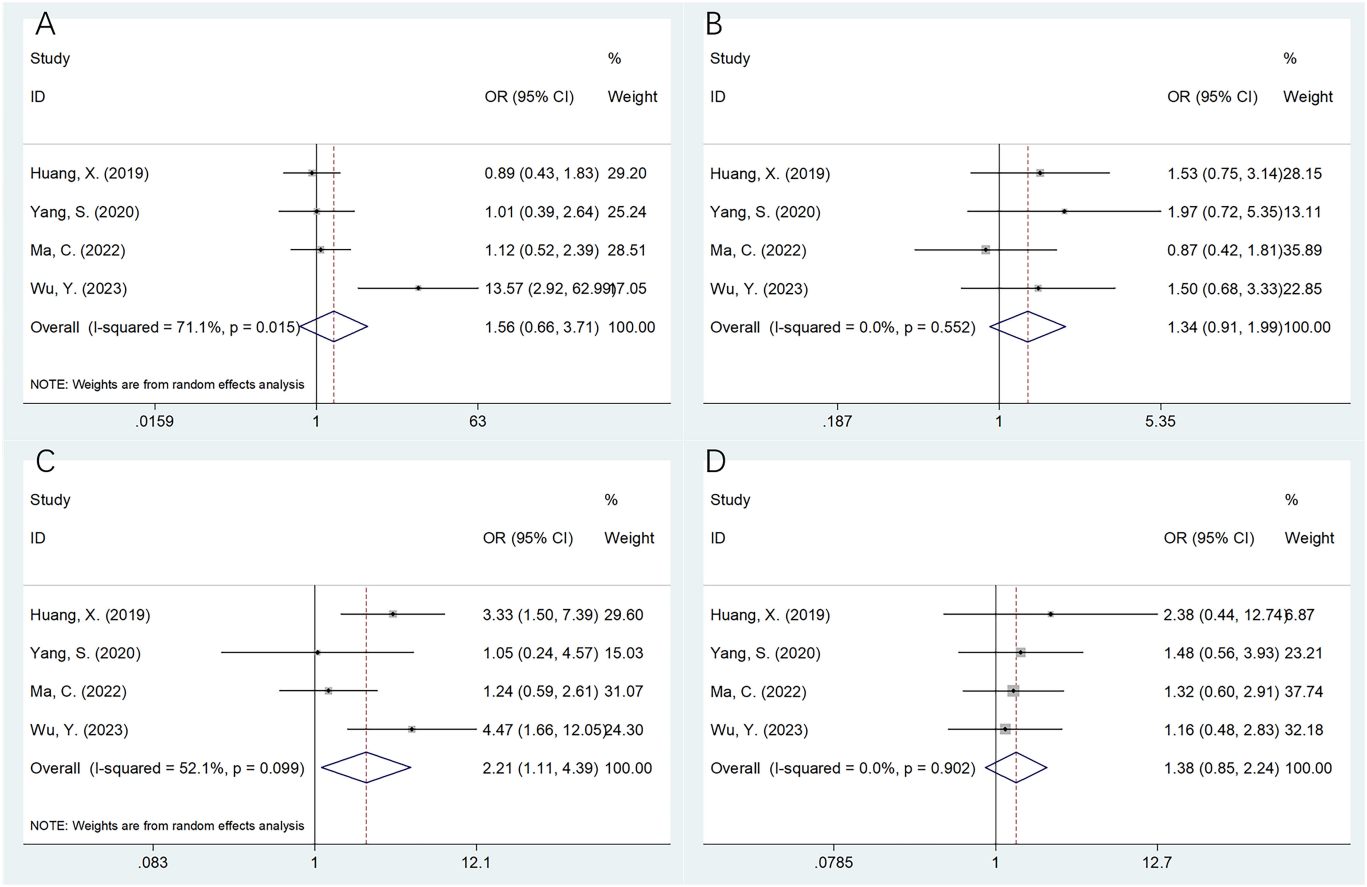
Forest plots assessing the relationship between the SII and clinicopathological factors in OSA. **(A)** Gender (male vs female); **(B)** Age (years) (≥20 vs <20); **(C)** Enneking stage (III vs I-II); and **(D)** Pathological fracture (yes vs no).

**Figure 4:**
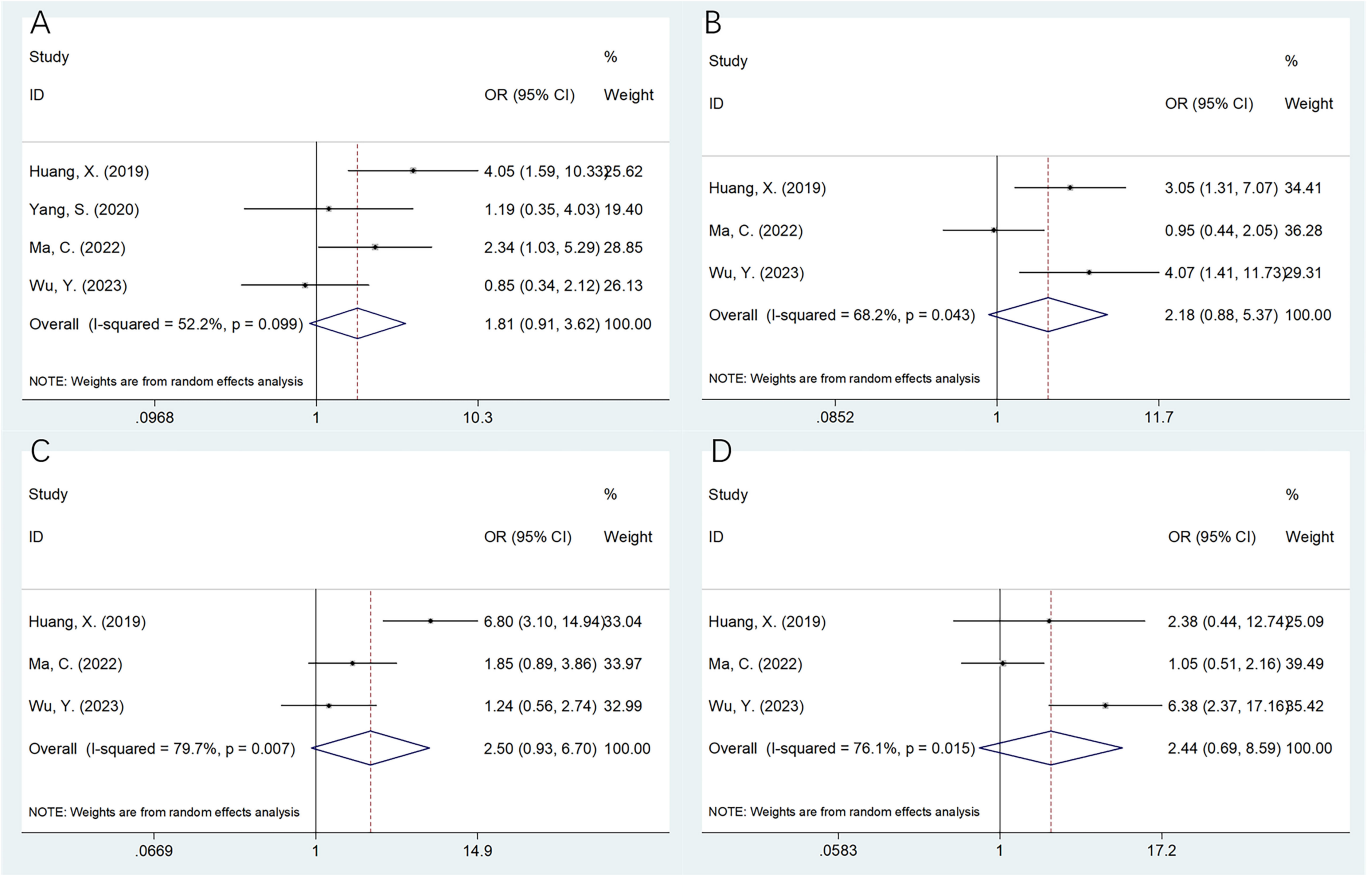
Forest plots assessing the relationship between the SII and clinicopathological factors in OSA. **(A)** Tumor location (non‐extremities vs extremities); **(B)** Tumor size (cm) (≥5 vs <5); **(C)** Tumor differentiation (poor vs well); and **(D)** Metastasis (yes vs no).

Four studies with 436 patients (20–22, 25) provided the data on association of SII with the OSA clinicopathological characteristics. According to pooled data, an elevated SII was remarkably related to Enneking stage III in patients with OSA (OR=2.21, 95%CI=1.11-4.39, p=0.024; **Table 3**; **Figure 3**). However, SII was not significantly related to gender (OR=1.56, 95%CI=0.66-3.71, p=0.315), age (OR=1.34, 95%CI=0.91-1.99, p=0.141), pathological fracture (OR=1.38, 95%CI=0.85-2.24, p=0.189), tumor location (OR=1.81, 95%CI=0.91-3.62, p=0.092), tumor size (OR=2.18, 95%CI=0.88-5.37, p=0.092), tumor differentiation (OR=2.50, 95%CI=0.93-6.70, p=0.069), and metastasis (OR=2.44, 95%CI=0.69-8.59, p=0.164) of OSA patients (**Table 3**; **Figures 3**, **4**).

**Table 3:** The association between SII and clinicopathological features in patients with osteosarcoma.

Factors	No. of studies	No. of patients	Effects model	OR (95%CI)	p	Heterogeneity	
						I^2^(%)	Ph
Gender (male vs female)	4	436	Random	1.56(0.66-3.71)	0.315	71.1	0.015
Age (years) (≥20 vs <20)	4	436	Fixed	1.34(0.91-1.99)	0.141	0	0.552
Enneking stage (III vs I-II)	4	436	Random	2.21(1.11-4.39)	0.024	52.1	0.099
Pathological fracture (yes vs no)	4	436	Fixed	1.38(0.85-2.24)	0.189	0	0.902
Tumor location (non‐extremities vs extremities)	4	436	Random	1.81(0.91-3.62)	0.092	52.2	0.099
Tumor size (cm) (≥5 vs <5)	3	359	Random	2.18(0.88-5.37)	0.092	68.2	0.043
Tumor differentiation (poor vs well)	3	359	Random	2.50(0.93-6.70)	0.069	79.7	0.007
Metastasis (yes vs no)	3	359	Random	2.44(0.69-8.59)	0.164	76.1	0.015

### Sensitivity analysis

**Figure 5:**
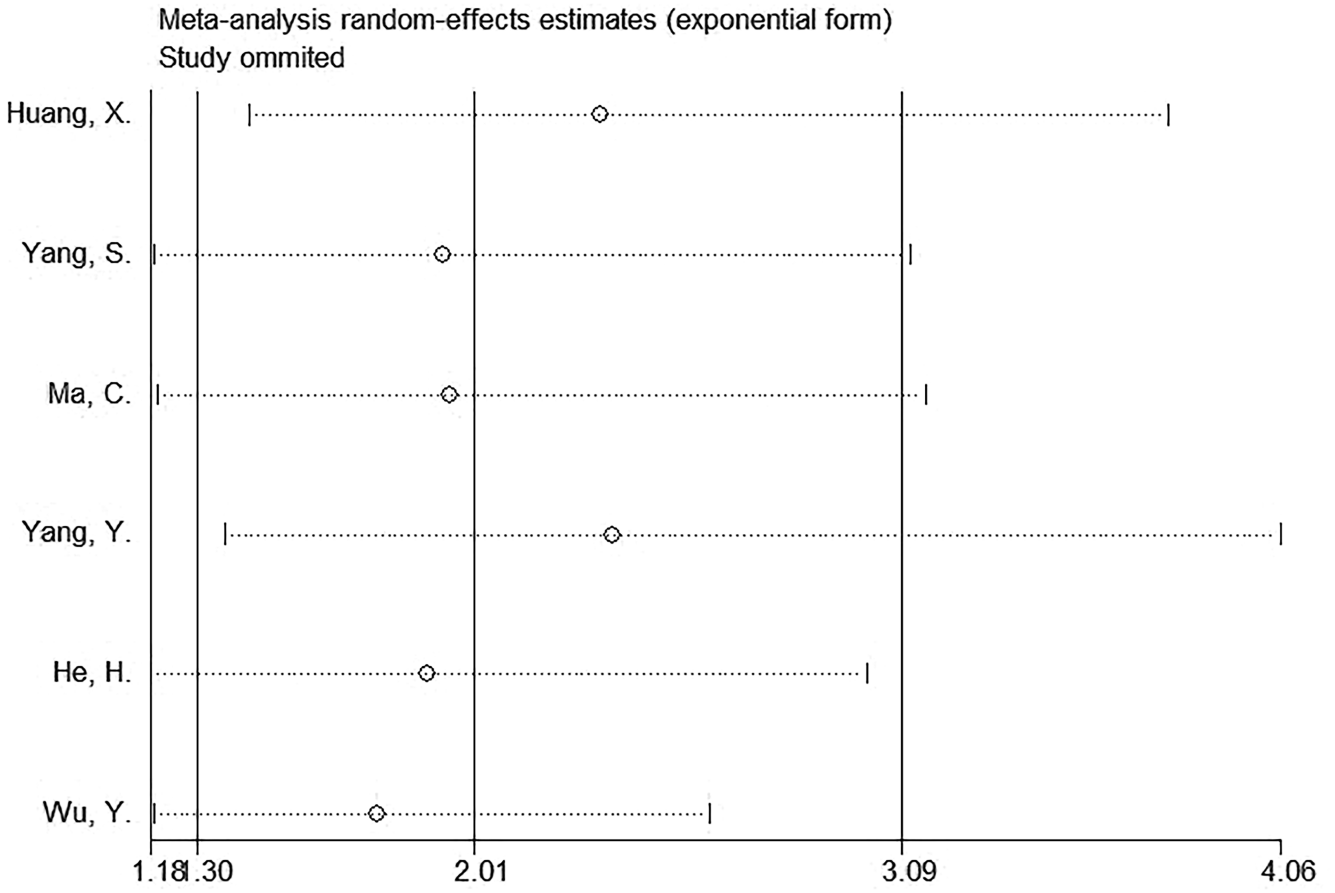
Sensitivity analysis for OS.

Sensitivity analysis suggested that the observed effect size for the relationship of SII with OS was not affected by any single study (**Figure 5**), which indicated the stability of the results in this work.

### Publication bias

**Figure 6:**
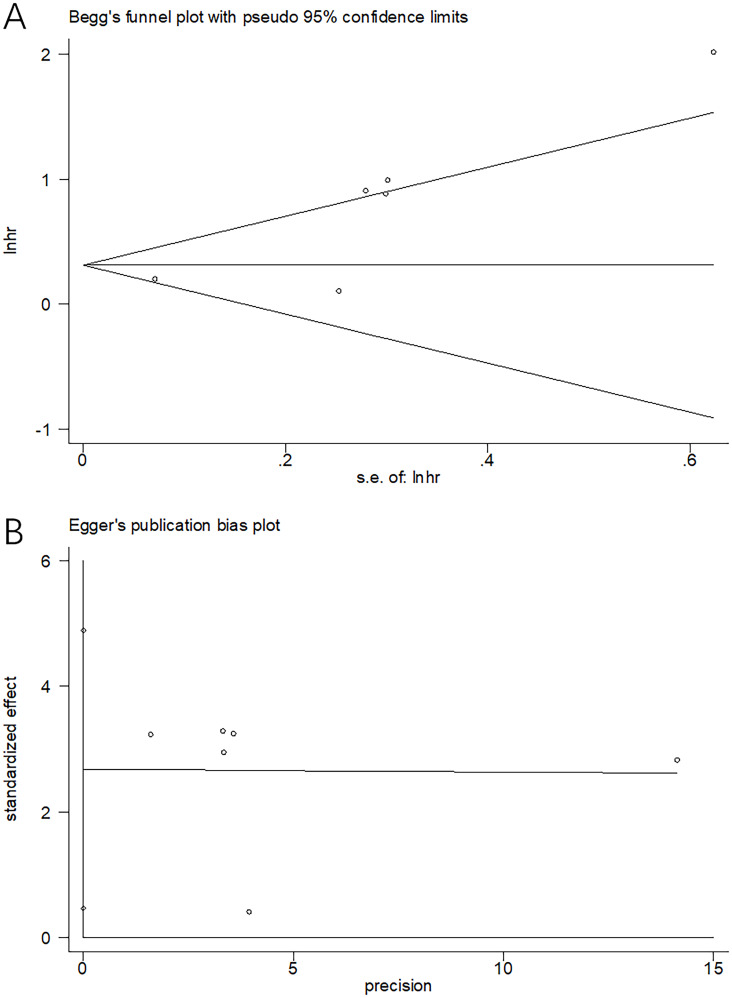
Publication bias tests. **(A)** Begg’s test for OS, p=0.124; **(B)** Egger’s test for OS, p=0.178.

We employed Begg’s test and Egger’s test for evaluating the possible publication bias. From **Figure 6**, funnel plots showed symmetry. Moreover, the results (p=0.124 and 0.178 upon Begg’s and Egger’s tests separately) demonstrated the absence of significant publication bias (**Figure 6**).

## Discussion

According to prior reports, the effect of SII on predicting prognosis of OSA patients remains controversial. In this work, information was synthesized in six articles involving 1015 patients for identifying the precise effect of SII on predicting OSA prognosis. In this meta-analysis, an elevated SII significantly predicted OS of OSA patients. Moreover, the higher SII was markedly related to advanced Enneking stage in OSA. SII served as the cheap and reliable prognostic marker for OSA patients. As far as we know, this meta-analysis is the first to investigate the effect of SII on predicting OSA prognosis.

The higher SII is the result of higher platelet number, higher neutrophil number, and/or lower lymphocyte number. The precise mechanisms of the association between SII and prognosis of OSA are not fully elucidated yet. Notably, the value of SII in predicting OSA prognosis is interpreted from several aspects. First, several studies have reported that platelets may protect cancer cells from the immune system’s cytotoxicity (29). In tumor cells, cytokines may stimulate megakaryocytes to produce platelets, resulting in an increase in platelet count (30). Platelets can also facilitate epithelial-mesenchymal transition (EMT) in cancer cells by directly contacting them or indirectly secreting prostaglandin E2 and growth factors (29). Second, during tumorigenesis, neutrophils contribute to the proliferation, invasion, and migration of tumor cells as well as tumor immunosuppression (31). Through the secretion of chemokines and cytokines, neutrophils are able to directly affect tumor cells or have an indirect effect on other tumor microenvironment (TME) components (32). These include vascular endothelial growth factor, transforming growth factor-beta, matrix metalloproteinases, interleukin-6 (IL-6), and IL-8 (33). Third, lymphocytes are crucial to anti-tumor cell-mediated responses. Lymphocytes can migrate into the TME and evolve into tumor-infiltrating lymphocytes (TILs), which can suppress the proliferation and migration of tumors through apoptosis (34, 35). Therefore, a higher SII can serve as the reasonable marker for predicting OSA prognosis.

Recently, SII is widely reported in meta-analysis with significant value in predicting prognosis of different solid tumors (36–40). According to Yang et al. in their meta-analysis involving 30 studies, the higher SII levels before treatment were related to poor OS and recurrence−free survival (RFS) of gastric cancer (36). Zhang and colleagues reported in their meta-analysis with 3464 patients that the higher SII was remarkably related to poor OS and DFS, low differentiation degree and advanced stage of oral squamous cell carcinoma (37). As mentioned in one meta-analysis enrolling 8133 patients with prostate cancer, a high SII was dramatically associated with poor OS, and worse progression-free survival/biochemical recurrence-free survival (PFS/bRFS) (38). In the meta-analysis comprising 2169 patients, Zeng et al. reported that the higher SII served as the effective marker to predict OS and PFS of nasopharyngeal carcinoma (39). Based on a meta-analysis with 1402 patients, the high SII was related to dismal OS of cholangiocarcinoma patients undergoing invasive surgery (40). Our meta-analysis findings were consistent with those in other cancer types. Notably, tumor necrosis rate is an important index for cancer treatment in patients with OSA (41, 42). A tumor necrosis rate > 90% usually indicates necrosis of tumor tissue, which showed inhibition of the blood supply to tumor tissue (41). In this meta-analysis, the association between SII and tumor necrosis rate was not analyzed due to limited information in included studies. The relationship between SII and tumor necrosis rate should be explored in future studies.

There were some limitations in the present work. First, this study had a small sample size. We just recruited six studies with 1015 patients, although we searched the most recent literature and did not restrict publication language. Second, all included studies were from China. In this regard, the findings in this work are more applicable for Chinese OSA populations. While the value of SII in the prognosis of OSA in other regions needs to be explored. Third, only retrospective studies were enrolled, which might lead to selection bias. Fourth, we only analyzed the prognostic value of SII for OS in this meta-analysis. The association between SII and other survival endpoints such as RFS, PFS, and DFS etc. was not investigated for OSA patients. We actually did not exclude RFS, PFS, and DFS in eligibility criteria, they were not included because of limited data provided in eligible studies. The correlation between SII and RFS, PFS, and DFS in OSA needs to be explored in future studies. Considering the above limitations, large-scale multi-regional prospective studies should be conducted for validation.

## Conclusions

In summary, according to our meta-analysis results, the higher SII is remarkably related to poor OS of OSA patients. Additionally, the elevated SII is also significantly related to advanced Enneking stage in OSA. SII is the candidate prognostic marker of OSA.

## Data Availability

The original contributions presented in the study are included in the article/**Supplementary Material**. Further inquiries can be directed to the corresponding author.
