# Supplementary material for: Prognostic and clinicopathological value of systemic immune-inflammation index in patients with osteosarcoma: a meta-analysis
Source: Front Immunol. 2024 Aug 15;15:1416068. doi: 10.3389/fimmu.2024.1416068 (PMC11357927; doi:10.3389/fimmu.2024.1416068)
Supplement: Supplementary file 1 [file Table1.docx]

**Supplemental file 1. The detailed search strategies for each database in this meta-analysis.**

1. **Search strategies for PubMed:**

Search: (systemic immune-inflammatory index or SII or systemic-immune-inflammation index or systemic immune-inflammation index) and (osteosarcoma or osteogenic sarcoma or bone sarcoma) Sort by: Most Recent

((("systemic"[All Fields] OR "systemically"[All Fields] OR "systemics"[All Fields]) AND "immune-inflammatory"[All Fields] AND ("abstracting and indexing"[MeSH Terms] OR ("abstracting"[All Fields] AND "indexing"[All Fields]) OR "abstracting and indexing"[All Fields] OR "index"[All Fields] OR "indexed"[All Fields] OR "indexes"[All Fields] OR "indexing"[All Fields] OR "indexation"[All Fields] OR "indexations"[All Fields] OR "indexe"[All Fields] OR "indexer"[All Fields] OR "indexers"[All Fields] OR "indexs"[All Fields])) OR ("stat interface"[Journal] OR "sii"[All Fields]) OR ("systemic-immune-inflammation"[All Fields] AND ("abstracting and indexing"[MeSH Terms] OR ("abstracting"[All Fields] AND "indexing"[All Fields]) OR "abstracting and indexing"[All Fields] OR "index"[All Fields] OR "indexed"[All Fields] OR "indexes"[All Fields] OR "indexing"[All Fields] OR "indexation"[All Fields] OR "indexations"[All Fields] OR "indexe"[All Fields] OR "indexer"[All Fields] OR "indexers"[All Fields] OR "indexs"[All Fields])) OR (("systemic"[All Fields] OR "systemically"[All Fields] OR "systemics"[All Fields]) AND "immune-inflammation"[All Fields] AND ("abstracting and indexing"[MeSH Terms] OR ("abstracting"[All Fields] AND "indexing"[All Fields]) OR "abstracting and indexing"[All Fields] OR "index"[All Fields] OR "indexed"[All Fields] OR "indexes"[All Fields] OR "indexing"[All Fields] OR "indexation"[All Fields] OR "indexations"[All Fields] OR "indexe"[All Fields] OR "indexer"[All Fields] OR "indexers"[All Fields] OR "indexs"[All Fields]))) AND ("osteosarcoma"[MeSH Terms] OR "osteosarcoma"[All Fields] OR "osteosarcomas"[All Fields] OR ("osteosarcoma"[MeSH Terms] OR "osteosarcoma"[All Fields] OR ("osteogenic"[All Fields] AND "sarcoma"[All Fields]) OR "osteogenic sarcoma"[All Fields]) OR ("osteosarcoma"[MeSH Terms] OR "osteosarcoma"[All Fields] OR ("bone"[All Fields] AND "sarcoma"[All Fields]) OR "bone sarcoma"[All Fields]))

Translations

systemic: "systemic"[All Fields] OR "systemically"[All Fields] OR "systemics"[All Fields]

index: "abstracting and indexing"[MeSH Terms] OR ("abstracting"[All Fields] AND "indexing"[All Fields]) OR "abstracting and indexing"[All Fields] OR "index"[All Fields] OR "indexed"[All Fields] OR "indexes"[All Fields] OR "indexing"[All Fields] OR "indexation"[All Fields] OR "indexations"[All Fields] OR "indexe"[All Fields] OR "indexer"[All Fields] OR "indexers"[All Fields] OR "indexs"[All Fields]

SII: "Stat Interface"[Journal:__jid101471232] OR "sii"[All Fields]

index: "abstracting and indexing"[MeSH Terms] OR ("abstracting"[All Fields] AND "indexing"[All Fields]) OR "abstracting and indexing"[All Fields] OR "index"[All Fields] OR "indexed"[All Fields] OR "indexes"[All Fields] OR "indexing"[All Fields] OR "indexation"[All Fields] OR "indexations"[All Fields] OR "indexe"[All Fields] OR "indexer"[All Fields] OR "indexers"[All Fields] OR "indexs"[All Fields]

systemic: "systemic"[All Fields] OR "systemically"[All Fields] OR "systemics"[All Fields]

index: "abstracting and indexing"[MeSH Terms] OR ("abstracting"[All Fields] AND "indexing"[All Fields]) OR "abstracting and indexing"[All Fields] OR "index"[All Fields] OR "indexed"[All Fields] OR "indexes"[All Fields] OR "indexing"[All Fields] OR "indexation"[All Fields] OR "indexations"[All Fields] OR "indexe"[All Fields] OR "indexer"[All Fields] OR "indexers"[All Fields] OR "indexs"[All Fields]

osteosarcoma: "osteosarcoma"[MeSH Terms] OR "osteosarcoma"[All Fields] OR "osteosarcomas"[All Fields]

osteogenic sarcoma: "osteosarcoma"[MeSH Terms] OR "osteosarcoma"[All Fields] OR ("osteogenic"[All Fields] AND "sarcoma"[All Fields]) OR "osteogenic sarcoma"[All Fields]

bone sarcoma: "osteosarcoma"[MeSH Terms] OR "osteosarcoma"[All Fields] OR ("bone"[All Fields] AND "sarcoma"[All Fields]) OR "bone sarcoma"[All Fields]

**2. Search strategies for Web of Science:**

(systemic immune-inflammatory index or SII or systemic-immune-inflammation index or systemic immune-inflammation index) and (osteosarcoma or osteogenic sarcoma or bone sarcoma) (all fields)

The link for this search strategy is as follows:

https://www.webofscience.com/wos/woscc/summary/713fdd94-ddb0-4036-aa58-41c4d1104c26-fe7a49da/relevance/1

**3. Search strategies for Embase:**

(systemic immune-inflammatory index or SII or systemic-immune-inflammation index or systemic immune-inflammation index) and (osteosarcoma or osteogenic sarcoma or bone sarcoma)

**4.** **Search strategies for Cochrane Library:**

(systemic immune-inflammatory index or SII or systemic-immune-inflammation index or systemic immune-inflammation index) and (osteosarcoma or osteogenic sarcoma or bone sarcoma) in Title Abstract Keyword

**5. Search strategies for CNKI:**

SII and 骨肉瘤

免疫炎症指数 and 骨肉瘤
